# Gold Added to Copper Alloy

**Published:** 1892-10

**Authors:** J. E. Register

**Affiliations:** Dover, Del.


### GOLD ADDED TO COPPER ALLOY.

BY J. E. REGISTER, D.D.S., DOVER, DEL.

In chemical reactions the resulting compound is always entirely different in its characteristics from any of its component parts; and this fact has strengthened me in the belief that there is great advantage in adding gold to copper alloy, to strengthen the latter in its resistance to the attacks on it by substances in the mouth,—many supposing these to be either sulphur, forming a soluble sulphate of copper, or acetic acid, forming a soluble acetate of copper. My theory leans more to the latter, as I believe acetic acid is the more readily formed in the mouth, owing to the acetic fermentation of the ingesta lodged in and between the teeth.

Dr. H. C. Register, of Philadelphia, suggested to me this treatment of copper alloy; but as it is yet a theory, I hope every operator reading this article will give it a trial, and if happy results follow, one of the best antiseptic filling-materials will be saved to dentistry. I will give my manner of preparing and inserting the alloy. The mercury is first prepared by placing in a small glass vial one ounce of the best redistilled mercury, and to this is added one leaf of No. 4 adhesive or soft gold-foil; shake the vial thoroughly for fully five minutes, and at the expiration of this period the gold will be found to be thoroughly dissolved. In this state the mercurialized gold (the name I have given it) can be kept any length of time, and is always ready for use. Then take an ingot of pure copper alloy, the requisite size to fill your cavity, and hold it over an alcohol flame till small beads of mercury cover the entire surface of the ingot. Then place it in a mortar and grind with pestle till of a smooth, soft, pasty mass; place this mass in a chamois-skin, winding it up tight, and, with the assistance of heavy, flat-nosed plyers, squeeze all the free mercury out. Place this flat, dry cake in a mortar and grind with pestle, adding to it enough mercurialized gold to make a soft, pasty mass. Take this mass and squeeze one-half of it between the finger and thumb to the consistency of stiff putty; take the other half and wind tight in a chamois-skin, and with plyers squeeze very dry. Fill the cavity first with the softer mass, and as the free mercury comes to the surface, add small pieces of the dry, till all the free mercury is taken up and the filling is solid and firm. Then burnish and instruct the patient not to disturb it in any way for at least from twelve to twenty-four hours. Now, as to whether this gold added will meet with the results desired will take several months to thoroughly test. "In the multitude of counsel there is wisdom," and with the numerous readers of this journal there may be many who can suggest some way of better manipulation. If so, I, for one, would gladly hear from them; and also hope many will give the suggestion a trial.

